# Genetic and molecular bases of esophageal Cancer among Iranians: an update

**DOI:** 10.1186/s13000-019-0875-4

**Published:** 2019-08-31

**Authors:** Mohammad Reza Abbaszadegan, Vahideh Keyvani, Meysam Moghbeli

**Affiliations:** 1 Medical Genetics Research Center, Mashhad University of Medical Sciences, Mashhad, Iran; 2 Department of Biology, Faculty of Science, Shahid Chamran University of Ahvaz, Ahvaz, Iran

**Keywords:** Esophageal cancer, Diagnosis, Panel marker, Prognosis, Iran

## Abstract

**Abstract:**

**Background:**

Esophageal cancer is one of the leading causes of cancer related deaths among the Iranians. There is still a high ratio of mortality and low 5 years survival which are related to the late onset and diagnosis. Majority of patients refer for the treatment in advanced stages of tumor progression.

**Main body:**

It is required to define an efficient local panel of diagnostic and prognostic markers for the Iranians. Indeed such efficient specific panel of markers will pave the way to decrease the mortality rate and increase the 5 years survival among the Iranian patients via the early diagnosis and targeted therapy.

**Conclusion:**

in present review we have reported all of the molecular markers in different signaling pathways and cellular processes which have been assessed among the Iranian esophageal cancer patients until now.

## Background

Esophageal cancer is the fifth common cancer in developing countries with a five-year survival less than 5%. It has a variable prevalence based on geographical districts from 20/100,000 in the US to 160/100,000 in China. Esophageal cancer belt with almost 60% of esophageal cancers in the world spreads from North of Iran and Kazakhstan to certain areas of China [1–4]. Genetic predisposition could potentially be effective in esophageal cancer progression. Most of studies in Iran and China showed that ESCC outbreak is double as much higher among close relatives of ESCC patients than among those lacking familial history [5]. Several polymorphisms related to the ESCC were also identified in three populations of Golestan province with high risk, Ardebil with medium risk, and Zoroastrians with low risk [6]. And finally during a study in Turkmen and Non-Turkmen Iranian populations, it was found that there was not an outstanding difference between Turkmens and Non-Turkmens from the point of genetic predisposition to ESCC. However, the average impact of genetic factors could not be completely rejected. More studies are required to investigate environmental and genetic risk factors of ESCC in Golestan province [7]. There is a high risk region for esophageal ESCC at Northeast of Iran where the previous studies have shown that a series of hereditary factors are involved in high incidence of this cancer in the region. Therefore, the genetic variations could be considered as a genetic predisposition in increasing or decreasing the probability of ESCC in Iran [8]. Since there is not a complete genetic panel of ESCC pattern among the Iranian population, we have tried to report all of the genetic and molecular markers which are involved and observed among the Iranian esophageal cancer patients in the current review (Table 1). In this regard we categorized all of the reported markers in different cellular processes.

**Table 1:** All of the involved markers in esophageal cancer susceptibility among the Iranian patients

Study (et al)	Year	Gene	Purpose	Population	Results
salehi [9]	2006	eIF4E	Prognosis	99 N/T^a^	Over expression
MOGHBELI [10]	2014	MSI1	Diagnosis	53 N/T	Correlation with sex
KAHKHAIE [11]	2014	MUC1	Prognosis	50 N/T	Correlation with stage, grade, and lymph node
SEDIGHI [12]	2016	MMP13	Diagnosis and Prognosis	66 patients 54 controls	Correlation with lymph node, tumor size, and survival
golyan [13]	2019	MMP21	Diagnosis and Prognosis	58 N/T	Correlation with stage
SALEHI [14]	2016	BRUCE	Diagnosis	50 N/T	Correlation with stage
ABBASZADEGAN [15]	2008	P16	Diagnosis	52 patients 50 controls	Hypermethylation
taghavi [16]	2010	P16	Diagnosis	50 N/T	Hypermethylation
ABBASZADEGAN [17]	2005	P16	Diagnosis	30 patients 30 controls	Hypermethylation
abedi-ardekani [18]	2011	P53	Diagnosis	119 patients	Mutation
khalilipour [19]	2018	KCNJ12	Diagnosis	9 patients	Mutation
forghanifard [20]	2019	DIDO1	Diagnosis	50 N/T	Correlation with depth of invasion
Ghobadi [21]	2018	CDKN2A	Prognosis	123 patients	Polymorphism was correlated with ESCC risk
hashemi-bidokhti [22]	2017	MAML1	Diagnosis and Prognosis	56 N/T	Over expression was correlated with depth of invasion, grade, stage, and sex
forghanifard [23]	2012	MAML1, TWIST1	Diagnosis and Prognosis	55 N/T	Over expressions were correlated with lymph node metastasis and stage
MOGHBELI [24]	2013	EGFR, PYGO2	Diagnosis and Prognosis	55 N/T	Correlation with stage and grade
FORGHANIFARD [25]	2014	SALL4, SOX2	Diagnosis	50 N/T	Correlation with lymph node and depth of invasion
MAHMOUDIAN [26]	2017	Cripto-1	Diagnosis	50 N/T	Correlation with tumor grade, stage, and location
MALLAK [27]	2016	EVX1	Diagnosis	50 N/T	Correlation with lymph node and depth of invasion
forghanifard [28]	2018	SIZN1	Diagnosis	50 N/T	Correlation with depth of invasion and lymph node metastasis
ANSARI [29]	2016	miR-93	Diagnosis	30 N/T	Over expression
ANSARI [29]	2016	miR-143	Diagnosis	30 N/T	Under expression
GHASEMI [30]	2018	miR-371–373	Diagnosis	36 N/T	Over expression
sahebi [31]	2016	Linc-ROR	Diagnosis	30 N/T	Correlation with grade
maghsudlu [32]	2019	miR-27a	Diagnosis and Prognosis	30 N/T	Over expression
AKBARI [33]	2008	BRCA2	Diagnosis	197 patients 254 controls	Mutation
forghanifard [34]	2011	MAGE-A4	Diagnosis and Prognosis	41 N/T	Over expression

^a^Tumor tissues and normal margins

## Main text

### Post transcriptional regulators

Eukaryotic initiation factors (eIFs) are involved in initiation of translation. The cap is detected by eIF4F and eIF4A unwinds the 5′ untranslated region using a helicase activity. EIF4E over expression enhances the synthesis of growth factors and oncogenes such as cyclin D1, FGF-2, VEGF, and c-myc. The eIF4E-binding proteins (4E-BPs) also mediate eIF4F complex formation through the prevention of interaction between eIF4E and eIF4G. It has been shown that the eIF4E expression was higher in tumors in comparison with normal margins among a sub population of Iranian esophageal cancer patients. The T4N1M0 tumors had highest levels of eIF4E expression [9]. Similarly, the eIF4E over expression was also observed in ESCC tissues of Chinese patients which were correlated with lymph node involvement and survival [35]. Posttranscriptional regulation is involved in different cellular functions such as cell development and growth. Musashi1 (MSI1) is involved in translational regulation as an inhibitor via a specific sequence located in the 3′ UTR of target mRNA [36]. It functions as an antagonist of teIF4G to bind with PABP [37]. P21WAF-1, Numb, and DKK3 are among the well-known MSI1 target factors which are involved in cell cycle regulation and WNT/NOTCH signaling pathways [36, 38]. It has been shown that there are not any significant correlations between MSI1 expression and clinicopathological features; however there was a significant correlation between sex and the levels of MSI1 mRNA expression. Therefore, MSI1 can be introduced as a sex dependent stem cell marker in ESCC cases [10]. In contrast, it has been reported that there was increased expression of MSI1 in Chinese ESCC samples compared with normal margins. Moreover, there were significant correlations between stage, lymph node involvement, and the levels of MSI1 expression [39].

### Extra cellular matrix and matrix metalloproteases

Hyaluronic acid and Laminin as non-collagenous components of the extracellular matrix are involved in cell adhesion and proliferation [40]. Levels of serum hyaluronic acid and Laminin were assessed among Iranian ESCC patients showing a higher levels of both of them in upper gastrointestinal (UGI) compared with normal tissues [41]. Hyaluronan-positive cancer cells can be observed in poorly differentiated and metastatic tumors. Early stage stratified epithelial tumors may also have Hyaluronan overexpression. Generally, serum hyaluronic acid and Laminin over expressions in UGI cancers can be used for the early detection [42]. MUC1 is a specific glycoprotein in the apical cell surface of normal glandular epithelium [43]. MUC1 is involved in various biological processes including cell adhesion, mucus production, immunosuppression, and cellular signaling. It exerts its oncogenic role via regulation of P53 and b-catenin [11, 44]. Levels of nine MUC1 variants mRNA expression were assessed in tumor and corresponding normal margins in ESCC patients. There were correlations between expression of (MUC1/C, D, and Z) isoforms and advanced stages, less tumor differentiation, and lymph node metastasis. Higher levels of MUC1-B expression were significantly associated with tumors with less progressive behavior. Generally, it was shown that MUC1/C, D, and Z splice variants were correlated with tumor progression in ESCC [45]. In contrast, the Chinese patients with MUC1 low-expression had significantly longer survival compared with high-expression cases. The metastatic tumors had significantly higher levels of MUC1 expression compared with non-metastatic tumors. Moreover, MUC1 over expression was correlated with poor prognosis [46]. Matrix metalloproteinases (MMPs) are a family of endopeptidases involving in tumor metastasis by ECM degradation. They also release the ECM-bound proteins such as growth and angiogenesis factors [47]. MMP-2 and MMP-9 play critical roles in the early stages of tumor invasion through degradation of type IV collagen in basement membrane and VEGF activation [48]. Although, the role of MMP-2 (−1306C/T), MMP-9 (−1562C/T), and MMP-12 (−82A/G) polymorphisms were analyzed among Iranian ESCC patients, they were not useful prognostic markers in the identification of susceptible ESCC cases [49]. Another study has also shown that there was a significant higher level of serum MMP-13 in Iranian ESCC patients compared with normal subjects. Levels of MMP-13 were significantly correlated with lymph node metastasis, tumor size, and survival rates [12]. Another group has been also reported that the MMP-21 overexpression was significantly correlated with advanced stages of tumor [13]. Similarly, MMP-21 expression was significantly elevated in Chinese ESCC patients. Moreover, the levels of MMP-21 were significantly correlated with lymph node metastasis, TNM stage, and overall survival [50].

### Apoptosis and cell cycle

Apoptosis functions as a tumor suppressor through the elimination of cells with harmful mutations. BRUCE as one of the inhibitors of apoptosis proteins (IAP) suppresses the caspases 3, 6, 7, and 9 [51], and facilitates degradation of apoptotic proteins such as caspase-9 and SMAC/DIABLO [52, 53]. Nuclear and cytoplasmic expression of BRUCE has been observed in ESCC tissues. Advanced stage tumors had more levels of nuclear BRUCE protein expression in comparison with the normal squamous cells of the esophagus [14]. P53, p16INK4a, and MDM2 are essential G1 cell cycle regulators which are associated with tumor progression [54]. The p16 is an inhibitor of the cyclin D–depended protein kinase which is inhibitor of G1/S checkpoint [55]. Methylation of p16INK4a promoter sequence is a critical mechanism for its inactivation and promotes the ESCC progression [56]. P16 promoter hypermethylation has been observed among the ESCC cases in Northeastern Iran [15]. Methylation status of the p16 promoter was evaluated using a MSP assay in 50 ESCC patients. There was a significant correlation between p16 protein expression and methylation. Moreover, significant differences were observed in methylation and loss of p16 protein expression between tumor and normal tissues highlighting the critical role of epigenetic among Iranian ESCC cases [16]. P16 hypermethylation was observed in 73% of Iranian cases. Moreover, p16 hypermethylated ESCC patients had 59 and 36% of p16 hypermethylation in their blood and serum samples, respectively [17]. It has been shown that there was an association between p16 promoter methylation and poor prognosis in Japanese ESCC patients [57]. A mutation prevalence of 50% was observed among Iranian ESCC tumors which had significantly higher ratio of G: C to A: T transitions at CpG dinucleotides of P53 [58]. Golestan province has the highest rate of somatic TP53 mutations with at least one mutation in 89.9% of the cases. There was also a correlation between mutation patterns and tea drinking habits in which wild-type TP53 and G: C to A: T transitions were observed among people who drink tea urgently. Transversion mutations were also observed among patients who prefer to drink tea with a delay. Majority of these missense mutations results in accumulation of mutant p53 protein [18]. CpG transitions by deamination of 5-methylcytosine to thymine can be associated with chronic inflammation [59, 60]. Chronic inflammation also promotes tumorigenesis [61]. Generally, rates of CpG dinucleotides mutations shows that there is a probable correlation between inflammatory process and ESCC progression in Iran. The p53 is employed to protect cells against DNA-damaging agents such as cigarette smoking [62]. The p21 protein as a DNA synthesis inhibitor is regulated by wild type p53 [63]. To evaluate the probable correlation between P53/P21 and cigarette smoking, levels of p53 and p21 protein expressions were assessed among 80 Iranian ESCC patients. They observed p53 and p21 as important players in ESCC progression in Northeast of Iran in which accumulation of abnormal p53 in normal margins can be a predictor of tumor recurrence. Moreover, they have shown a converse correlation between survival and p21 expression among the ESCC cases. Therefore, p21/p53 protein expression can be introduced as useful prognostic markers among Iranian patients with poor clinical outcome [64]. The p53 overexpression was correlated with tumor differentiation, tumor size, and overall survival in a sub population of Chinese ESCC patients [65]. In contrast, there were not any correlation between p53 expression and clinicopathological features and prognosis among a group of Japanese ESCC cases [66]. In the case of Iranian familial cases, previous reports have shown that the first-degree relatives had higher risks of ESCC in comparison with the relatives of unrelated controls (34% vs. 14%) [33, 67]. Whole-Exome Sequencing (WES) was employed to assess the germ line mutations in nine Iranian ESCC families. Three out of four detected variants included amino acid changes within KCNJ12/KCNJ18 gene. KCNJ12 encodes a potassium channel protein controls the tumor growth by cell cycle arrest. Moreover, KCNJ12 is associated with NF-kB signaling pathway [19]. We have also assessed the role of DIDO1 as an activator of Caspase 9 and 3 during the apoptosis process in a sample of Iranian ESCC patients. There was a significant correlation between DIDO1 expression and tumor depth of invasion. It was shown that the DIDO1 has an important role in primary stages of tumor progression [20]. The CDKN2 is also a regulator of cell cycle. It has been shown that there was a correlation between rs10811661 and rs1333049 in CDKN2A/B loci and poor prognosis in Iranian ESCC patients in which the CC genotype carriers had a lower survival rates [21].

### NOTCH/WNT signaling pathways

NOTCH signaling is a cell to cell contact pathway which is contributed with angiogenesis, differentiation, and self-renewal [68]. Therefore, aberrant NOTCH pathway can be observed in developmental malignancies and cancers. NOTCH receptors and ligands binding on adjacent cells release the NOTCH intracellular domain (NICD) into the cytoplasm. NOTCH transcriptional machinery involves NICD, DNA-binding protein CSL (CBF1/Su (H)/LAG-1), and MAML1–3 co activators [69, 70]. It has been reported that there was a higher levels of MAML1 protein expression in tumors compared with normal ESCC tissues. Although, there was not any MAML1 expression in normal margins, 59% of tumor samples showed MAML1 protein overexpression. There were also significant correlations between MAML1 expression, sex, grade, depth of tumor invasion, and stage of ESCC samples. These results highlighted the MAML1 as a possible metastatic marker among Iranian ESCC cases [22]. TWIST1 is an oncogenic transcription factor in EMT process. It has been suggested that the TWIST1 enhances metastatic ability of tumor cells in ESCC [71]. MAML1 and TWIST1 over expressions have been reported among a sub population of Iranian ESCC samples in which aberrant expressions of MAML1/TWIST1 were correlated with depth of invasion, node metastasis, and surgical stage [23]. The TWIST1 expression in stromal fibroblasts was assessed in Korean ESCC patients which was positively correlated with increased depth of tumor invasion, lymph node involvement, stage, and overall survival [72]. In another study on Korean ESCC cases, the TWIST1 expression was associated with poor prognosis and EMT in ESCC tissues [73]. Deregulation of WNT signaling pathway is commonly observed in malignancies [74]. Activation of canonical WNT signaling results in cytoplasmic b-catenin accumulation, consequently LEF/TCF-1 complex activation. PYGO2 is also one of the components of LEF/TCF-1 complex which binds to the H3K4me to activate the transcription process [75]. EGFR is a tyrosine kinase involving in activation of various cellular processes such as cell proliferation, differentiation, and tumor progression [76]. EGFR and PYGO2 mRNA expressions were assessed in tumor and corresponding normal margins in a sample of Iranian ESCC cases. EGFR/PYGO2 overexpression had significant correlations with surgical stage and tumor grade. There was also inverse correlations between EGFR expression, lymph node involvement, and tumor depth of invasion. Moreover, there was a significant correlation between PYGO2 and EGFR indicating the PYGO2 as a transcriptional activator of EGFR expression in ESCC cases [24]. There are different associations between WNT and NOTCH pathways in ESCC progression. We have reported a significant correlation between MSI1 and PYGO2 and tumor depth of invasion in ESCC cases [77]. In another study we have also shown that there wre significant correlations between MAML1 and PYGO2 as the main components of NOTCH and WNT signaling transcriptional machineries respectively and tumor depth of invasion and size among a group of Iranian ESCC cases [78]. EGFR expression was significantly associated with TNM staging, lymph node involvement, and distant metastasis among a sub population of Chinese ESCC cases [79]. There were also significant correlations between nuclear expression of EGFR, TNM stage, lymph node metastasis, and poor prognosis in a group of Japanese ESCC patients [80].

### Self-renewal factors

ESCC cancer stem cells (CSCs) are a subpopulation of tumor cells with ability of self-renewal and chemo- and radio-therapeutic resistance. Regulatory transcriptional networks which govern CSCs maintenance and progression introduce novel therapeutic targets for elimination of CSCs. SALL4 and SOX2 are transcription factors associated with self-renewal and pluripotency of embryonic stem cells [81, 82]. It was reported that there was a significant correlation between SALL4 and SOX2 in ESCC tissues. SOX2/SALL4 over expression was associated with lymph node metastasis and depth of tumor invasion, introducing these factors as important players through the tumorigenesis from beginning to metastasis in ESCC patients. Their data confirm the importance of CSCs in ESCC progression among the Iranian patients [25]. In contrast, it has been shown that the high SOX2 expression was correlated with negative lymph node metastasis in Taiwanese ESCC cases [83]. Cripto-1 (CR-1) is a member of the EGF-CFC gene family and plays a critical role in self-renewal, differentiation and cell migration via MAPK and AKT intracellular signaling pathways [84, 85]. Moreover, OCT-4 and NANOG are the regulators of CR-1 [86]. CR-1 overexpression was reported in a wide spectrum of solid tumors [87]. CR-1 overexpression was observed in 38% of ESCC samples. There were significant correlations between CR-1 expression and tumor grade, stage, and location. Therefore, CR-1 is introduced as an oncogene during the ESCC progression and metastasis among the Iranian patients [26]. Similarly, it has been observed that there were associations between the levels of CR-1 expression, TNM stage, depth of invasion, and lymph node involvement in a sample of Chinese ESCC patients [88].

### BMP pathway and HOX genes

BMP signaling is associated with different cellular processes such as proliferation, differentiation, and tumor invasion [89]. EVX1 is shown as a target gene in BMP signaling pathway. Levels of EVX1 were assessed in ESCC patients showing a significant correlation between the low levels of EVX1 expression, lymph node metastasis, and depth of tumor invasion [27]. ZCCHC12 (SIZN1), as a transcriptional co-activator of BMP signaling, is identified as a positive regulator of central nervous system development during embryogenesis. It positively regulates the CREB and AP1 transcription factors which are in cooperation with BMP signaling pathway. We have recently assessed the levels of SIZN1 mRNA expression in ESCC patients. There were significant correlations between SIZN1 expression and lymph node involvement and tumor depth of invasion, introducing the SIZN1 as an important factor in early stages of tumor progression among Iranian ESCC cases [28]. MEIS1 transcription factor is a member of the three amino acid loop extension (TALE) family of homeodomain proteins and is involved in self-renewal [90]. It was reported that MEIS1 expression has an inverse correlation with lymph node involvement and tumor stage in ESCC [91]. Moreover, It was shown that MEIS1 as one of the HOX members is associated with WNT and NOTCH pathways through the MSI1. We have recently reported a significant correlation between MEIS1/MSI1 over expression and depth of tumor invasion, in which the over expressed cases were observed among the tumors with higher depths of invasion [92]. On the other hand, MEIS1 can be functioned as a negative regulator for the NOTCH pathway among the Iranian ESCC patients [93].

### Non coding RNAs

MicroRNAs (miRNAs) are noncoding small RNAs regulating different cellular processes including cell proliferation and differentiation through a post transcriptional repression [94]. MiR-143 has a tumor suppressive role [95, 96] via inhibition of KRAS and ERK5 [96, 97]. It has also reported that the miR-93 can be involved in tumor metastasis through repressing LATS2 and p21 expression [98, 99]. Levels of miR-143 and miR-93 have been assessed in tumor and normal margins of Iranian ESCC cases showing a significant increase and decrease of miR-93 and miR-143 expression in tumors compared with normal tissues, respectively. However, there was not any significant correlation between such markers and clinicopathological features of ESCC patients [29]. Similarly, the levels of miR-143 expression was significantly decreased in ESCC tissues compared with normal margins in Chinese cases. Moreover, there was an inverse association between miR-143, depth of invasion, and lymph node involvement [100]. MiR-371–373 regulate self-renewal of cancer stem cells through WNT signaling pathway in various tissues [101]. P53, RRBP1, and LATS2 tumor suppressors are the target genes of miR-371–373 cluster [102]. Expression of miR-371–37 cluster has been assessed in Iranian ESCC samples and demonstrated the miR-371–373 over expression in tumors compared with normal margins. It was suggested that the miR-371–373 over expression can be involved in tumorigenesis through cell cycle regulators such as CDK2, CDK4, and CDK6 [30]. MiR-451 is another important regulator of cell proliferation, migration, and apoptosis which is related to different factors such as CAB39 [103], RAB14 [96], and MIF [104]. Circulatory miR451 has been introduced as a diagnostic marker of esophageal cancer. Malignant fibroblasts secrets miR-451 into their environment and prepare a suitable condition for the aggressive tumor cells. It has been shown that although there was miR-451 over expression in ESCC serum, the tumor samples had miR-451 under expression compared with normal tissues [105]. Long non-coding RNAs (lncRNAs) are one of the main players in regulation of cellular functions including proliferation, self-renewal, chromatin remodeling, and cell reprogramming [106, 107]. LincRNA-ROR is regulated by OCT4, SOX2, and Nanog. Linc-ROR prevents degradation of miR-205 target genes such as ZEB2 through the EMT process [108]. It also suppresses P53 post transcriptionally, and prevents the cell cycle arrest [109]. Pattern of linc-ROR splice variants (2 to 5) expression were assessed in Iranian ESCC cases, showing an over expression of variants 2 and 4 in tumors compared with normal margins. There was also a significant correlation between variant 4 and grade of tumors [31]. SOX2OT is known as SOX2 LncRNA and has been reported to participate in regulation of SOX2. Therefore, a study assessed the SOX2OT expression in ESCC samples, introducing the SOX2OT splice variant during ESCC progression and metastasis [110]. MiR-27a is one of the regulators of WNT signaling through SFRP1 targeting [111]. The miR-27a over expression was observed among a sub population of Iranian ESCC patients [32].

### DNA repair

BRCA2 is one of the members of homologous recombination repair and is also involved in various cellular functions such as cell proliferation [112] and cell cycle regulation [113]. Germline DNA of 197 Turkmen ESCC cases have been assessed for the BRCA2 mutations. A deletion of 13 nucleotides at position 501–513 and a stop codon (UAA) at codon 91 were identified. A single nucleotide deletion at position 3734 leads to a truncated BRCA2 protein lacking DNA repair domains [33]. Microsatellite instability (MSI) is one of the major involved defects in neoplastic transformation [114]. These repeated DNA sequences are widely observed within the genome leading to the variations among individuals [115]. MSI is an indicator of deficient mismatch repair (MMR) system [116]. It was shown that there is a significant correlation between LOH and MSI and clinicopathological features of Iranian ESCC patients [117]. Loss of MLH1 and/or MSH2 expressions as the MMR components significantly were associated with metastatic lymph nodes and poorly differentiation among a group of Japanese ESCC cases. Moreover, the MLH1-negative cases significantly had poorer prognosis compared with MLH1-positive cases [118].

### Cancer-testis antigens

Cancer-testis antigens (CTAs) are mostly expressed in placenta and gonads in normal condition [119]. However, aberrant CTAs expressions have been reported in a variety of cancers. MAGE-A4 is associated with transcriptional control via binding to the PIAS2, in which the c-terminal of MAGE-A4 inhibits transcription through interactions with HDAC1 and SNW1 [120]. MAGE-A4 prevents MIZ-1 to bind with p21 promoter resulting p21-mediated cell cycle arrest [121]. MAGE-A4 overexpression has reported in more than 90% of ESCC patients, and was correlated with lymph node metastasis and advanced stages of tumor. Correlation of MAGE-A4 with LAGE1 and NY-ESO1 in tumor progression, demonstrated that the CTAs are efficient options for the targeted therapy in ESCC cases [34]. In contrast, there were not any significant correlation between MAGE-A expression, TNM classification, and grading among a sample of German ESCC patients. Moreover, MAGE-A expression had not any prognostic significance [122]. Another study on Chinese ESCC patients revealed that there was a significant MAGE-A9 up regulation in ESCC tissues compared with normal. Moreover, the levels of MAGE-A9 expression was significantly correlated with grade, size, and lymph node involvement [123].

## Conclusion

Iran is located on the Asian esophageal cancer belt which spreads from China to Northern Iran. Therefore, Iran is among the countries with a high EC incidence and mortality. Regarding the lack of an efficient molecular early detection method, majority of patients refer for treatment at advanced stages of tumor progression which significantly decreases the efficiency of treatments. Therefore it is required to introduce a population based panel markers for the early detection of EC in this population. In present review we summarized all of the reported genetic markers which were involved in EC progression among Iranian patients. Moreover, we categorized the reported genes based on their cell and molecular functions to clarify the biology of EC in this population. Indeed this review paves the way of determination of a population based panel of diagnostic markers among Iranian EC patients.

## Data Availability

The datasets used and/or analyzed during the current study are available from the corresponding author on reasonable request.

## Abbreviations

4E-BPs: EIF4E-binding proteins
CR-1: Cripto-1
CSCs: Cancer stem cells
CTAs: Cancer-testis antigens
eIFs: Eukaryotic initiation factors
ESCC: Esophageal squamous cell carcinoma
IAP: Inhibitors of apoptosis proteins
lncRNAs: Long non-coding RNAs
miRNAs: MicroRNAs
MMPs: Matrix metalloproteinases
MMR: Mismatch repair
Msi1: Musashi1
NICD: Notch intracellular domain
TALE: Three amino acid loop extension
TGF-β: Transformation growth factor beta
WES: Whole-Exome Sequencing
